# The use of Mitomycin-C to reduce synechia in middle meatus in sinus surgery: preliminary results

**DOI:** 10.5935/1808-8694.20120007

**Published:** 2015-11-20

**Authors:** Wellington Yugo Yamaoka, Luís Carlos Gregório

**Affiliations:** a MSc. Sciences (Assisting Physician in the Rhinology Sector of the Otorhinolaryngology Course at the University of São Paulo); b PhD (Head of the Otorhinolaryngology Course at the University of São Paulo). Universidade Federal de São Paulo

**Keywords:** mitomycin, natural orifice endoscopic surger, postoperative complications

## Abstract

Synechia is the most frequent complication after sinus surgery and has been reported in up to 36% of cases. Several types of materials have been used to reduce the incidence of synechia, including Mitomycin C (MMC).

**Objective:**

This prospective study aimed to assess the effectiveness of topical MMC in the prevention of synechia after sinus surgery in humans.

**Methods:**

At the end of surgery, MMC solution (1.0 mg/ml) was topically applied randomly to one of the middle meatuses (MMC group) of 14 patients while saline solution was applied to the contralateral meatus (control group). The author remained blind to the medicated side. Synechiae were classified as partial or total.

**Results:**

Three patients had middle meatus synechia in the MMC group (21.43%) *versus* nine (64.29%) in the control group (*p* = 0.054). In the MMC group, all three middle meatus synechia were partial, while in the control group there were four partial (28.57%) and five total (35.71%) cases of synechia (*p* = 0.025).

**Conclusions:**

Mitomycin C was not effective in preventing middle meatus synechia, but reduced the probability of total synechia formation.

## INTRODUCTION

The treatment of choice for refractory chronic rhinosinusitis is functional endoscopic sinus surgery (FESS), one of the most frequently performed procedures in the ENT practice. Despite its efficacy and safety, 7.6% to 38% of the patients experience relapsing symptoms and undergo revision surgery^1^^,^^2^. The causes of treatment failure include disturbed mucociliary clearance, immunodeficiency, sensitivity to acetylsalicylic acid, and anatomic obstruction. The most frequent events related to the latter cause are synechia and ostium stenosis (27% in maxillary sinuses and 25% in frontal sinuses)^3^^,^^4^.

Postoperative synechia is the most frequently reported complication in the literature, found in between 11% and 36% of the cases. Revision surgery is required in 1% to 2% of the cases^5^, ^6^, ^7^, ^8^. When only revision surgery is considered, the incidence rate ascends to 56%^4^. A review on 182 FESS patients revealed that the only findings related to little clinical improvement were fibrosis in medial antrostomies and in the ethmoidal region^3^.

A number of different materials has been tried to reduce the rate of complications, among which are Merocel®, FloSeal™, Sepragel® and hyaluronic acid^9^. Mitomycin C (MMC) has also been studied as a means to prevent postoperative fibrosis.

MMC is an antibiotic-antineoplastic agent first isolated from *Streptomyces caespitosus* in 1958^10^. It inhibits the synthesis of DNA through a bifunctional alkylation that leads to the crossing of double helical strands not allowing neoplastic cells to proliferate. Higher dosages of MMC also inhibit the synthesis of RNA and proteins, and it has been used for many years now in oncology care. It inhibits scarring when used topically^11^.

The antiproliferative effect MMC has on human fibroblasts is the reason why it can modulate scarring and prevent excessive fibrosis formation. It has antiproliferative properties at 0.04mg/ml and, in higher dosages, offers cytocidal effects^9^^,^^10^.

One single topical application of MMC for 5 minutes offers antiproliferative effects for up to 36 hours^9^. When briefly applied to the human mucosa it reduces the replication of fibroblasts and increases their apoptosis^10^.

MMC was first used topically in 1963 as adjuvant therapy for pterygium^11^, reducing relapse rates from 89% to 2.3%^12^. In glaucoma surgery it has proven effective in preventing stenosis of the trabeculectomy drainage fistula^13^. Additionally, it has improved the outcomes of dacriocistorhinostomy^14^^,^^15^.

MMC has been used more recently in ENT procedures such as laryngotracheal stenosis repairs^16^, to maintain patency in myringotomy^17^^,^^18^, and its use in sinus surgery has been successfully studied^19^, ^20^, ^21^, ^22^.

Ingrams et al.^19^, in a trial with rabbits, looked into patency, antrostomy areas, and ciliary function of the mucosa treated with MMC dosages of 0.04 mg/mL, 0.4 mg/mL and 1 mg/mL. The authors verified that 0.4 mg/mL and 1 mg/mL of MMC maintained patency and antrostomy areas with a statistically significant difference against controls. Additionally, ciliary appearance and function returned to normal within two weeks and and the mucosal surface re-epithelialized normally. Rahal et al.^20^, using 1 mg/mL of MMC in rabbits, concluded the drug maintained antrostomy patency in areas significantly greater than saline solution without complications. However, studies in human beings with 0.4 mg/mL and 0.5 mg/mL of MMC failed to replicate the outcomes seen in experimental trials^23^^,^^24^.

This paper aimed to assess the effectiveness of mitomycin C in humans to prevent synechia after functional endoscopic sinus surgery in humans.

## METHOD

This study was approved by the Research Ethics Committee and granted permit n° 0191/04. The sample consisted of 15 patients (eight males and seven females) aged between 20 and 67 years (mean age of 42.79 years) with chronic rhinosinusitis and referred to surgical treatmentsof Otorhinolaryngology. Patients were asked to sign an informed consent term. Inclusion criteria: diagnosis of chronic rhinosinusitis as defined by the Consensus of the Brazilian Association of Otorhinolaryngology; age between 18 and 70 years; capacity to understand and sign the informed consent term; indication to undergo functional endoscopic sinus surgery. Exclusion criteria: pregnancy; primary cilia dyskinesia; cystic fibrosis; immunodeficiency; presence of nose or sinus tumor. One patient was excluded due to inadequate postoperative follow-up. The extent of disease was determined based on paranasal sinus CT scans and scored in accordance with the Lund-Mackay staging system.

All patients were operated under general anesthesia by the author from May to October of 2006. Seventy-seven separate procedures were carried out. Three patients (21.43%) had allergic rhinitis, one (7.14%) had asthma, and one (7.14%) had systemic hypertension. Two patients (21.43%) had been previously operated for chronic rhinosinusitis. One had an antrostomy with bilateral ethmoidectomy and a polypectomy respectively. The other had undergone two antrostomy procedures with ethmoidectomy.

In the beginning of the procedure the nasal cavities were filled with a 4×1 cm cotton swab soaked in 0.75% ropivacain with 1/100,000 UI epinephrine. Septal deviations hampering the access to ostiomeatal complexes were corrected at the start of the procedure. The extent and location of the disease determine which cavities would be approached.

Endoscopic surgery was done using 4-mm Karl Storz® 0°, 30° and 45° rigid scopes. The procedure was started with the placement of one surgical grade 2 × 1 cotton swab soaked in 1/1.000 UI epinephrine in each middle meatus. Then the middle concha insertion was injected with 0.75% ropivacain and 1/100,000 UI epinephrine solution.

The Messerklinger approach was used to treat the involved paranasal sinuses. Antrostomy procedures were done in the posteroanterior orientation in a non-circular fashion with a diameter always greater than 3 mm as defined by Stammberger. At the end of the procedure a surgical grade 2 × 1 cotton swab with 1 mL of MMC (1.0 mg/ml) was placed on one of the middle meatuses for 5 minutes. Another cotton swab soaked in 1 mL of saline solution was placed in the contralateral control middle meatus for 5 minutes. The cotton swabs were put in place by another team member based on a randomization protocol produced on an Excel spreadsheet to blind the author. The cotton swabs were then removed and the middle meatuses packed with 10 × 2 rayon soaked in topical bacitracin until the next morning. All patients were given antistaphylococcus antibiotics after surgery for seven days and were advised to wash their noses with 20 mL of saline solution six times a day.

Postoperative visits were done one and two weeks, and one, three, six, and 12 months after surgery. Patients were seen by the author, who remained blinded as to the group they belonged. The parameter assessed was presence of synechia in the middle meatus. Patients were inquired about pre and postoperative symptoms and endoscopically examined by the author using 4-mm Karl Storz® 0°, 30° and 45° scopes. Synechia was characterized as adhesion between mucosas and were categorizes as partial when the middle meatus was not completely obstructed and complete when they occluded them completely. Meatuses were deemed open only when no synechia was found.

Statistical analysis was done considering the scores assigned in the one-year postoperative visits, once they portrayed reality at the end of the study. The incidence rates of synechia in the middle meatuses in the groups were compared. The following statistical tests were used: λ^2^, sign test, Fisher's exact test, Wilcoxon signed-rank test, and z test.

## RESULTS

Seventy-seven different procedures were performed, namely: 30 antrostomies, 30 ethmoidectomies, eight sphenoidotomies, five frontal sinusotomies and four septoplasties. The mean Lund-Mackay score was 14.50. The left side mean score was 7.36 and the right side mean score was 7.14. In the sides administered MMC the mean score was 7.14 while controls had a mean score of 7.36. This difference was not statistically significant (sign test, *p* = 0.50).

Variables gender, previous surgery, presence of polyps before surgery, and allergic rhinitis did not alter the results (Table 1), and neither did MMC administration (Fisher's exact test, *p* = 0.18) (Table 2). The type of procedure performed did not alter the incidence rate of synechia (Table 3).

**Table 1:** Incidence rates of middle meatus synechia and patient characteristics.

Variable	Patients (n)	Synechia (n)	No synechia (n)	*p*
Male	7	5	9	0.44
Female	7	7	7	
Previous surgery	3	2	4	0.67
Polyps	4	2	6	0.40
Allergic rhinitis	3	3	3	1.00

**Table 2:** Incidence rates of synechia based on side of middle meatus.

		Synechia		*p*
Side	MMC	Control	Total	
Right	3	3	6	
Left	0	6	6	
Total	3	9	12	0.18

**Table 3:** Incidence rates of synechia based on performed procedure.

Procedure	Synechia	No synechia	Total	*P*
Antrostomy	12	16	28	0.57
Ethmoidectomy	12	16	28	0.57
Sphenoidotomy	3	5	8	0.73
Frontal sinusotomy	1	4	5	0.38
Septoplasty	1	3	4	0.62
Total	29	44	73	

MMC was administered into nine (64.29%) right and five left (35.73%) middle meatuses. A total of 12 cases of synechia (42.86%) were identified in ten of the 14 patients (z test, *p* = 0.57). Eight patients had synechia unilaterally and two bilaterally. In the eight cases of unilateral synechia, one patient had synechia in the side treated with MMC and seven had it in the control side as (z test, *p* = 0.07) (Table 4).

**Table 4:** Location of synechiae in 14 patients.

	Side of synechia			
Site of synechia	Patients (n)	MMC	Control	*p*
Unilateral	8	1	7	0.07
Bilateral	2			
None	4			
Total	14			

**Graph 1:**
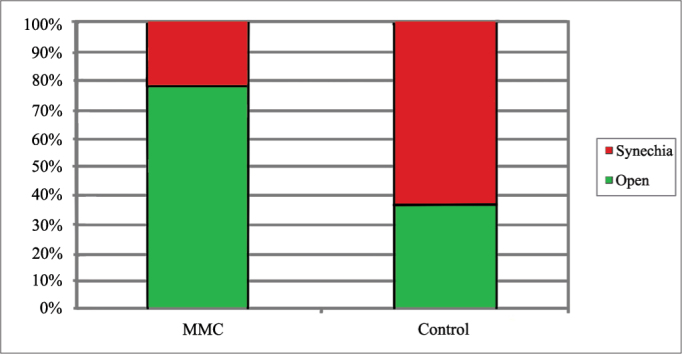
Incidence rates of middle meatus synechiae for each group.

One year after surgery, eleven (78.57%) middle meatuses treated with MMC and five (35.71%) in the saline solution group were free of synechia. Three middle meatuses in the MMC group (21.43%) and nine in the control group (64.29%) had synechia (Graph 1). There was no statistically significant difference between the groups (Fisher's exact test, *p* = 0.054).

**Graph 2:**
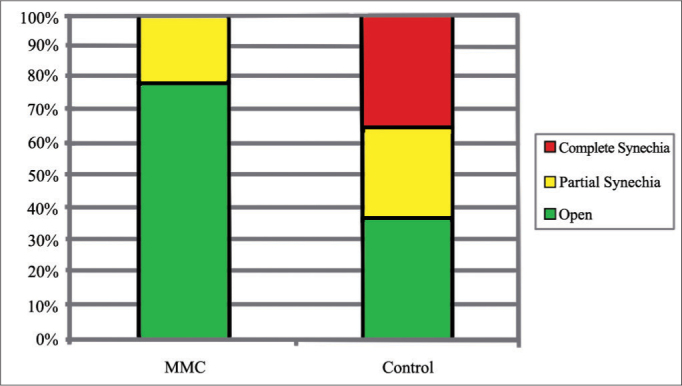
Incidence rates of middle meatus synechia types for each group.

When synechia cases are considered separately, according to our classification the MMC group had three cases (21.43%) of partial synechia and no cases of complete synechia, while the control group had four cases (28.57%) of partial and five patients (35.71%) with complete synechia. This difference was statistically significant (X^2^ test, *p* = 0.025) (Graph 2). Four patients had relapsing symptoms of rhinosinusitis in the control side. All had complete synechia, as opposed to the synechia-free side treated with MMC. None of the patients had to be re-operated, once symptoms subsided with clinical treatment.

All patients claimed they improved one year after surgery. Additionally, none of them reported adverse effects or required re-operation during the study. Four patients had polyps after surgery, two in the side treated with MMC and two in the side given saline solution. They had had polyps prior to surgery. One patient complained of recurring headaches before surgery and still had this symptom throughout the study, although he claimed to have improved from rhinorrhea and nasal obstruction. This patient specifically had had two procedures before the study and had polyps after surgery. Two patients had persistent thick nasal secretion in the control side which only improved after systemic administration of antibiotics. Two complications were observed: periorbital emphysema two weeks after surgery in one patient on the MMC-treated sideient.

## DISCUSSION

One of the main precautions taken during FESS is avoiding intracranial and orbital complications may have severe irreversible consequences. These can be mitigated with thorough knowledge of the local anatomy, surgical experience, and proper preoperative planning, so that our attention can be turned to trying to reduce postoperative complications such as synechia and stenosis.

According to the literature, synechia is the most frequent complication in functional endoscopic sinus surgery. It usually occurs between the middle concha and the lateral nasal wall^25^. The main cause of synechia is the non-preservation of the mucosa, allowing open areas to get in touch with and adhere to each other. Another less common cause is the presence of a pneumatized middle concha making it easier for the mucosa to touch the lateral nasal wall^26^.

The most effective measures to prevent the formation of synechia are preserving the mucosa during surgery and carrying out careful postoperative care, dressing the surgical wounds and offering outpatient care at the first sign of adhesion. In spite of these measures, some authors advocate the partial resection of the middle concha as a means to avoid complications^27^, ^28^, ^29^. Nonetheless, they still occur quite frequently.

We decided to study the effectiveness of topical MMC in reducing scarring and fibrosis formation in middle meatuses and antrostomy sites because this drug has been used successfully with no adverse effects for 40 years in pterygium and glaucoma procedures. Another factor that led to the choice of MMC was the fact that it has been used with equal success in other areas of ENT care.

In FESS, Ingrams et al.^19^ and Rahal et al.^20^ have shown that 0.4 mg/mL and 1 mg/mL MMC can prevent fibrosis in experimental models. In an attempt to demonstrate the same principle in humans, Chung et al.^23^ and Anand et al.^24^ carried out randomized controlled trials using MMC at 0.4 mg/mL and 0.5 mg/mL respectively, but no statistically significant differences were found when MMC was compared to saline solution. Nonetheless, as there was a tendency toward better results in patients treated with topical MMC, both authors suggested that higher dosages and other drug presentations should be tested.

Therefore, this study looked into the use of topical 1 mg/mL MMC administered in one single dose during surgery. Two complications occurred in our study: one case of periorbital subcutaneous emphysema 14 days after surgery on the side in which MMC had been administered with dehiscence of the lamina papyracea in the middle meatus not visualized during surgery; and an asymptomatic perforation of 0.8 cm in the posterior septum along its larger diameter in a patient submitted to septoplasty and sinus surgery.

The emphysema was probably caused by the surgical procedure, even though the dehiscence of the lamina papyracea was not seen during surgery, and not by the use of MMC. The patient recovered fully in one week. The perforated septum may have occurred because the perichondrium was not preserved during the septoplasty procedure.

The chosen method allowed the consideration of differences between sides exclusively due to MMC, once each patient served as their own control. Besides, there was no statistical difference in the procedures done on each side of the nose, between the mean Lund-Mackay scores, and patient characteristics. MMC administration was randomized and postoperative assessments were done by an examiner blinded for the side of MMC delivery.

The four patients with recurring symptoms had complete synechiae in their control meatuses and no synechia in their meatuses treated with MMC. This finding suggests that this type of synechia may have been a determining factor for synechia occurrence. Thus, synechiae were categorized as partial or complete. Given the efficacy of MMC in preventing the appearance of complete synechia, we may suggest that the drug led to a reduction on the recurrence of symptoms.

The rate of synechia in this study was 42.86% despite the slightly lower rates reported by other authors, which ranged between 11% and 36%. This was probably due to the fact that mucosal adhesions of any kind, including filiform tissue structures, were categorized as synechia in this study. However, the comparisons were made between enrolled subjects, and not between them and cases reported in the literature. Although there was no statistically significant difference between the groups, there was a clear tendency toward better results in the group treated with MMC.

When the complete synechia cases alone are considered, incidence rates dropped to 0% in the MMC group and 17.86% in the control group, to thus reflect the rates found in the literature.

At first, the increase on the MMC dosage to 1 mg/mL was not enough to prevent synechia formation, as reported by Chung et al.^23^ and Anand et al.^24^ for the 0.4 mg/mL and 0.5 mg/mL dosages respectively. On the other hand, unlike what both authors saw, we have proven that such increase in dosage is enough to prevent the occurrence of complete synechiae, one of the main symptom causing factors. Dosage and form of administration were not changed for that purpose.

The one-year follow-up period sat somewhere in the middle when compared to those used by Chung et al.^23^ and Anand et al.^24^ of four and 15 months respectively. The period of one year seemed to be appropriate, as most changes occurred within six months of follow-up.

We are aware that longer follow-up may reveal late complications and changes in outcome such as worsening of the meatuses treated with MMC. Given the significant time difference in the drug administration schedule of previous studies in relation to ours, future studies with intermediate dosages may be able to demonstrate the efficacy of the drug with higher safety levels.

The tendency toward better results with MMC despite the lack of statistical significance may be related to the small number of individuals enrolled in this study, vis-à-vis Chung et al.^23^ and Anand et al.^24^. Thus, given the high incidence rate of synechiae reported in the literature, an increase on the number of enrolled individuals may change the results reported in this study.

## CONCLUSION

Mitomycin C was not effective in preventing postoperative synechiae in patients submitted to functional endoscopic sinus surgery.

Mitomycin C was effective in preventing postoperative total synechiae in patients submitted to functional endoscopic sinus surgery.

Studies with larger populations, higher dosages, and longer time of exposure are required.
